# Enriched environment can reverse chronic sleep deprivation-induced damage to cellular plasticity in the dentate gyrus of the hippocampus

**DOI:** 10.1515/tnsci-2022-0280

**Published:** 2023-03-21

**Authors:** Xue Shixing, Hou Xueyan, Ren Yuan, Tang Wei, Wang Wei

**Affiliations:** Department of Neurology, Affiliated Xinhua Hospital of Dalian University, Dalian, Liaoning Province, China; Department of Medical Imaging, Affiliated Xinhua Hospital of Dalian University, Dalian, Liaoning Province, China; Department of Rehabilitation Medicine, Affiliated Zhongshan Hospital of Dalian University, Dalian, Liaoning Province, China

**Keywords:** Alzheimer’s disease, cellular plasticity, neurogenesis, enriched environment, chronic sleep deprivation

## Abstract

**Objective:**

We studied whether enriched environment (EE), a classic epigenetics paradigm, can prevent cellular plasticity damage caused by chronic sleep deprivation (SD).

**Methods:**

We performed SD in mice by a modified multi-platform method (MMPM). Mice in the SD group were deprived of sleep for 18 h a day. In addition, half of the mice in the chronic SD group were exposed to EE stimuli for 6 h per day. Immunostaining analyzed neurogenesis and neural progenitor cell-differentiated phenotypes in the hippocampal dentate gyrus (DG) region.

**Result:**

At 13 weeks, compared with the control group, SD severely impaired the proliferation and differentiation of neural stem cells, and EE completely reversed the process. SD can induce gliosis in the mouse hippocampus, and EE can delay the process.

**Conclusion:**

Our results suggest that chronic SD may damage the neurogenesis in the DG of the hippocampus. However, enrichment stimulation can reverse the processing by promoting neuronal repair related to neuronal plasticity.

## 1 Introduction

Alzheimer’s disease (AD) is the leading cause of dementia in the elderly [1]. As of 2018, the number of AD patients worldwide has exceeded 50 million [2]. The damage of AD is currently considered to be mainly related to the deposition of senile plaques, the formation of neurofibrillary tangles, and decreased brain plasticity [3]. Numerous studies have shown that amyloid-beta (Aβ) and Tau pathologies are directly related to impaired neurogenesis in AD [4–6]. These damaged neurons increase neural susceptibility and contribute to cognitive impairment [7].

Recent studies have shown that chronic sleep deprivation (SD) may be an independent risk factor for AD [8], especially in ordinary people and in preclinical AD stages [9]. Chronic sleep restriction increases soluble hippocampal Aβ and impairs cognitive performance [10–12]. The evidence suggests that long-term SD as a risk factor for AD can produce pathological changes similar to AD. Furthermore, Kreutzmann et al. found that chronic SD can impair brain neurogenesis and ultimately lead to reduced hippocampal volume [13]. It also means that similar to AD, cell plasticity, the ability of neural stem cells to differentiate into whole lineage-type cells under suitable conditions, may be an underlying mechanism of chronic SD.

Based on the earlier evidence, we hypothesized that chronic sleep disturbance could cause damage to cellular plasticity in the hippocampus. We established a sleep-deprived mouse model by a modified multi-platform method (MMPM) [14]. We evaluated neural stem-cell proliferation and the ability to differentiate into neurons and glial cells in the hippocampal dentate gyrus (DG) region.

In 1978, Rosenzweig et al. first proposed the definition of enriched environment (EE) as “a combination of complex inanimate and social stimulation” [15]. EE was originally proposed as a tool to study the effects of experience and behavioral activities on the brain. With further research on EE, this definition has gradually been refined. Compared to standard housing environments, EE places greater emphasis on motor, sensory, social, and cognitive stimuli (neural consequences of environmental enrichment). For a long time, EE as a classical paradigm is often used to study brain plasticity in post-stroke conditions [16], AD [17], Parkinson’s disease [18], and other neural degenerative diseases [19]. EE has always lacked a unified standard paradigm in animal or clinical experiments. However, some basic principles (complexity and novelty) and settings remain the same, including larger and more types of spaces, more animals, more changes (regular replacement of cages, built-in items, etc.), and more built-in items (running wheels, tunnels, mazes, brightly colored and differently shaped items, etc.). The mechanism of action of this model is extensive, involving the size and weight of the brain itself, gene expression, regulation of neurotransmitters, neurotrophic factors, hormones, and immune factors [20].

Although many experiments have shown that EE can enhance the proliferation and differentiation of brain’s neural stem cells, the relationship between long-term SD and nerve-cell plasticity is unclear, and whether the same benefits can be observed in SD models [21,22]. In order to further reveal the effects of EE, we established a chronic SD model to investigate the effect of EE on neuronal cell plasticity. Our study demonstrated that EE could reverse chronic SD-induced neurogenesis impairment in the DG region of mouse’s hippocampus and further explored its differentiation phenotype. We introduced the concept of EE into the chronic SD model for the first time, revealing the mechanism of long-term SD causing damage to cell plasticity and proving that EE may be an effective way to delay the pathological process.

## 2 Materials and methods

### 2.1 Animals

We randomly divided 5-month-old healthy male C57BL/6 J mice (Liaoning Changsheng Bio, 30–40 g) into a control group (CG), chronic SD group, and chronic sleep deprivation + EE group (SE). The laboratory temperature was controlled at 22 ± 1℃, and food and water were freely available.

### 2.2 Preparation of chronic SD model

**Figure 1:**
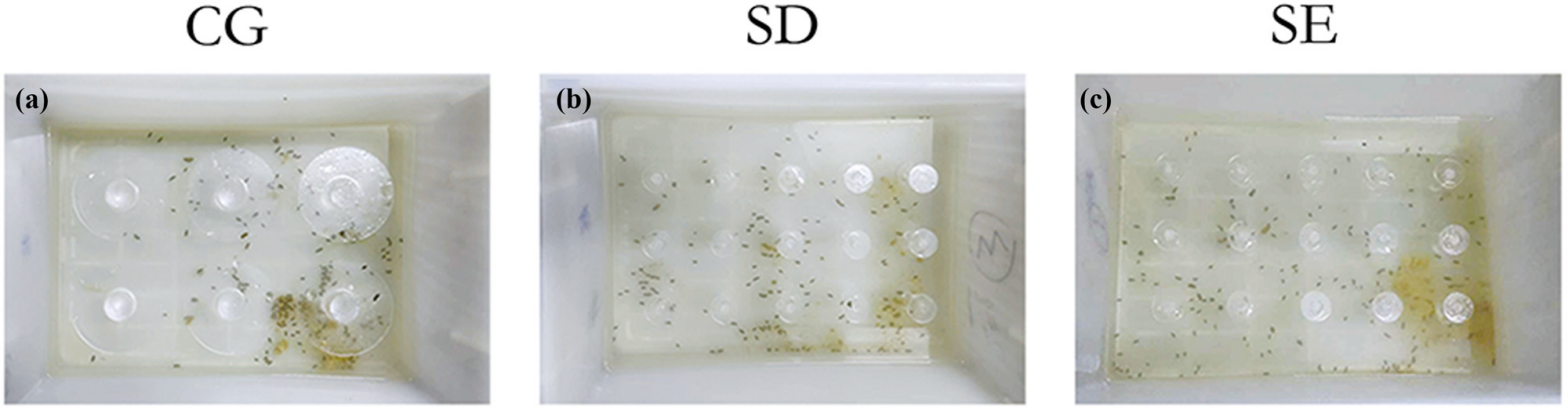
(a) The platform in the large platform water tank was large enough that the mice could rest and sleep without falling into the water. (b) and (c) In the small platform water tank, mice fell into the water due to muscle hypotonia during sleeping, forcing them to climb back onto the platform to stay awake. CG = control group; SD = sleep deprivation; SE = sleep deprivation + enriched environment.

We established the chronic SD model by the MMPM [14]. As shown in Figure 1, the SD and SE group involving small platforms is made of 39 cm × 27 cm × 20 cm opaque plastic. There are 15 platforms with a diameter of 3 cm and a height of 6 cm. The interval between the two platforms is about 4 cm, and the water level is controlled at 1–2 cm below the platform. Two mice were in each tank, and all mice had access to water and food overhead. During SD, mice fall into the water due to muscle hypotonia during sleeping, forcing them to climb back onto the platform to stay awake. MMPM deprived rapid eye movement (REM) sleep of mice. In the CG, a large platform water tank was used, and six platforms with a diameter of 10 cm and a height of 6 cm were placed at the bottom of each water tank, and the interval between the two platforms was about 2 cm. The difference between the two tanks was such that the platform in the CG was large enough that the mice could rest and sleep without falling into the water.

All mice were acclimated in a water tank 2 h a day before the start of the experiment for 3 days. After the start of the experiment, SD was performed for 18 h a day (4:00 PM–10:00 AM) for 12 weeks. The water in the tank was cleaned and replaced every day.

### 2.3 EE production

**Figure 2:**
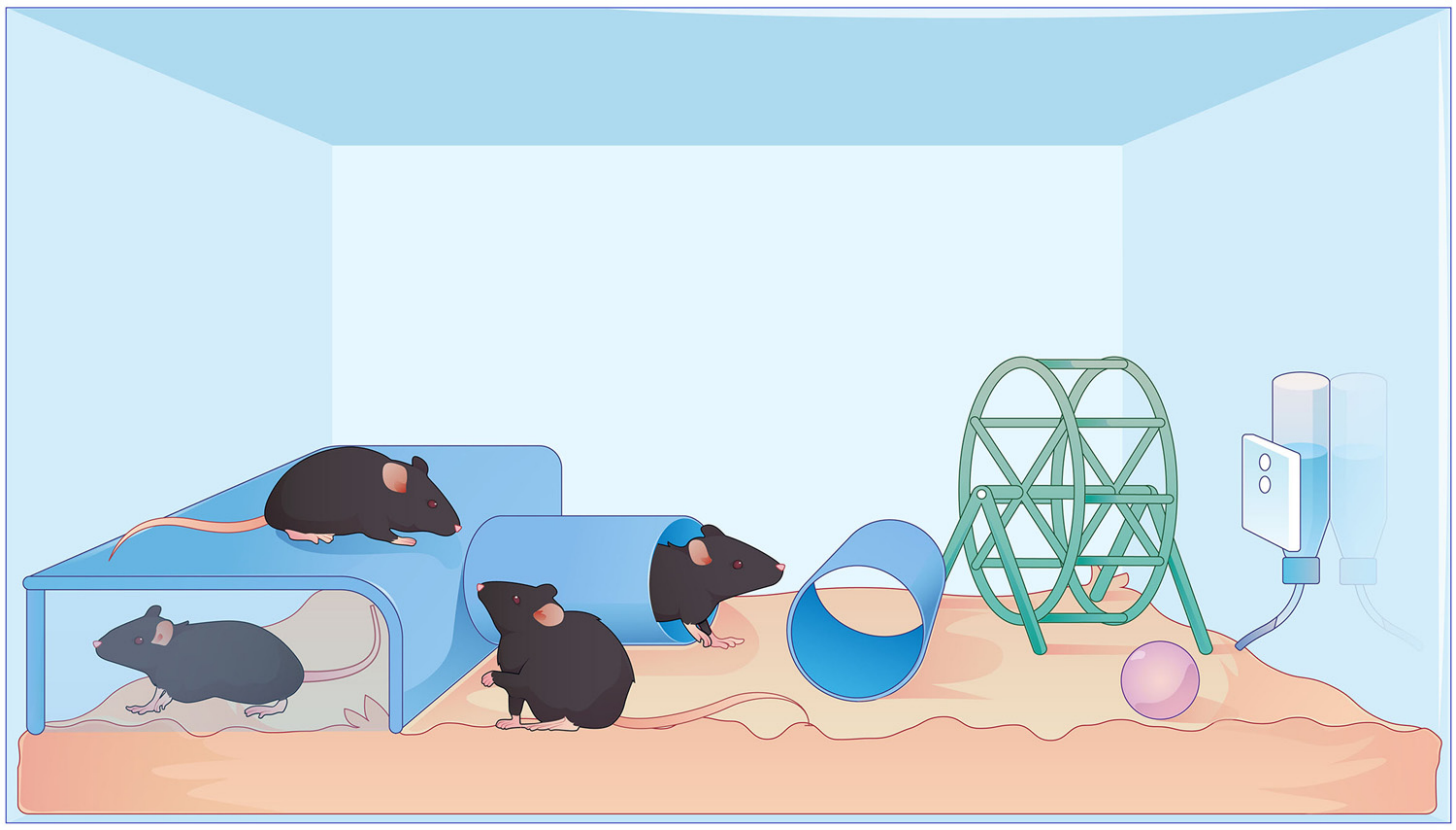
The elements of the enriched environmental paradigm. The EE intensified motor, sensory, cognitive, and social stimuli compared to the standard environment and followed some basic principles such as novelty and complexity.

In the standard environment, two mice per cage were kept in plastic mouse cages (32 cm × 17.5 cm × 14.5 cm), and only bedding was placed inside. The rich housing environment consists of larger rat cages (54 cm × 33 cm × 20 cm), with six mice in each cage. In addition to bedding, the cages are equipped with running wheels, geometric toys, labyrinth tunnels, and other items. To ensure novelty, cage objects and positions were changed weekly (Figure 2). After the start of the experiment, enrichment stimulation was performed for 6 h a day (10:00 AM–4:00 PM) for 12 weeks.

### 2.4 5-Bromo-2-deoxyuridine (BrdU) injection and tissue processing

After 12 weeks of intervention, we administered BrdU at 20 mg/mL (100 mg/kg, Sigma) in sterile saline intraperitoneally for five consecutive days [23,24]. Twenty-four hours after BrdU injection, all mice were anesthetized with 1% sodium pentobarbital (50 mg/kg, i.p.) and perfused with 50 mL of pre-cooled phosphate-buffered saline (PBS, pH 7.4) until the liver became white. The brains of mice were fixed with 4% paraformaldehyde, dehydrated with alcohol gradient, and made transparent with xylene. Then, according to the mouse brain atlas of Paxinos and Franklin “*The Mouse Brain in Stereotaxic Coordinates*,” the DG level representative area of the dorsal hippocampus was embedded in paraffin (1.22 mm/2.46 mm posterior–anterior to bregma). One hemibrain was serially cut into 4 μm thick sagittal sections using a paraffin microtome for immunostaining analysis (Leica RM2235, Leica Biosystems).

### 2.5 Immunohistochemistry

Five discontinuous coronal sections (4 μm thick) were randomly selected from each mouse for doublecortin (DCX) immunohistochemistry. The primary antibodies used were anti-DCX (1:250, ab207175, Abcam). The enzyme-labeled goat anti-rabbit IgG polymer was properly enhanced for incubation. 3,3′-Diaminobenzidin staining was performed for coloring, hematoxylin was counterstained, and pictures were taken using a Pannoramic DESK, P-MIDI (3D HISTECH, Hungary) scanner Image-Pro Plus 6.0 software analyzed the result of DCX, and all results were expressed as positive area ratios. We included sections without primary antibody incubation as negative controls to rule out possible false positives in the results.

### 2.6 Triple immunolabeling BrdU/NeuN/GFAP

Five discontinuous coronal sections were randomly selected in the hippocampal DG marker area (1.22 mm/2.46 mm posterior–anterior to bregma) for mature neuron markers (NeuN), astrocyte markers (glial fibrillary acidic protein, GFAP), and cell proliferation marker (BrdU) staining. The sections were dewaxed using xylene, dehydrated using an alcohol gradient, received antigen repairing with sodium citrate (PH = 6.0), blocked peroxidase with 3% H_2_O_2_, and blocked the sections with 10% goat serum at 37°C. The primary antibody was incubated at 4°C overnight, and the secondary antibody was incubated at 37°C for 1 h. TSA was incubated at 37°C for 30 min. After washing with PBS, the antigen retrieval steps, serum blocking, and antibody incubation were repeated. After the final antigen repair, nuclei were stained with 2-(4-Amidinophenyl)-6-indolecarbamidine dihydrochloride (DAPI). The primary antibodies were mouse anti-BrdU (1:100, GB12051, Servicebio), rabbit anti-GFAP (1:200, ab68428, Abcam), and rabbit anti-NeuN (1:2,000, ab177487, Abcam). The secondary antibodies were horseradish peroxidase (HRP)-conjugated goat anti-rabbit IgG (1:300, 074-1506, KPL) and HRP-conjugated goat anti-mouse IgG (1:300, 074-1806, KPL). The specific information of fluorescein is shown in Table 1. Pictures were taken using the Pannoramic DESK, P-MIDI (3D HISTECH, Hungary) scanner. Mean densitometric analysis of all stain results was performed using Image-Pro Plus 6.0 software. We manually removed slightly delaminated sites.

**Table 1:** Fluorescein information

Fluorescein	Maximum excitation wavelength (nm)	Maximum emission wavelength (nm)	Color under microscope	Picture color
TSA-520	490	520	Green	Green
TSA-570	550	570	Orange–red	Red
TSA-670	640	670	Red	Pink
TSA-480	430	480	Cyan	Cyan

### 2.7 Statistical analysis

We used the statistical software R version 3.5.3. All data are presented as mean ± SD. We first conducted normality and homogeneity of variance tests. The comparison of data between multiple groups conforming to normal distribution and homogeneity of variance was performed by GraphPad Prism analysis of variance for statistical analysis, and corresponding charts were drawn. If not, the rank sum test (*H* test) was used. Results were considered statistically significant when *P* < 0.05. *Post-hoc* analysis was performed by the Tukey–Kramer test.


**Ethical approval:** The research related to animals’ use has been complied with all the relevant national regulations and institutional policies for the care and use of animals. Animals were handled following the Animal Research: Reporting of In Vivo Experiments (ARRIVE) guidelines. Ethical approval for the use of mice was given by the Animal Ethics Committee of Affiliated Xinhua Hospital of Dalian University (2021-088-01).

## 3 Results

### 3.1 EE reverses chronic SD-induced neurogenesis damage beyond the CG

To evaluate the effect of EE on neurogenesis, we performed immunohistochemistry on the neurogenesis marker DCX. We calculated the positive area ratio of the DG area. As shown in Figure 4, DCX mainly includes irregular cell bodies located at the junction of the granule cell layer (GCL) and subgranular zone (SGZ) in the DG area and synapses extending to the molecular layer (Figure 4d–f).

**Figure 3:**
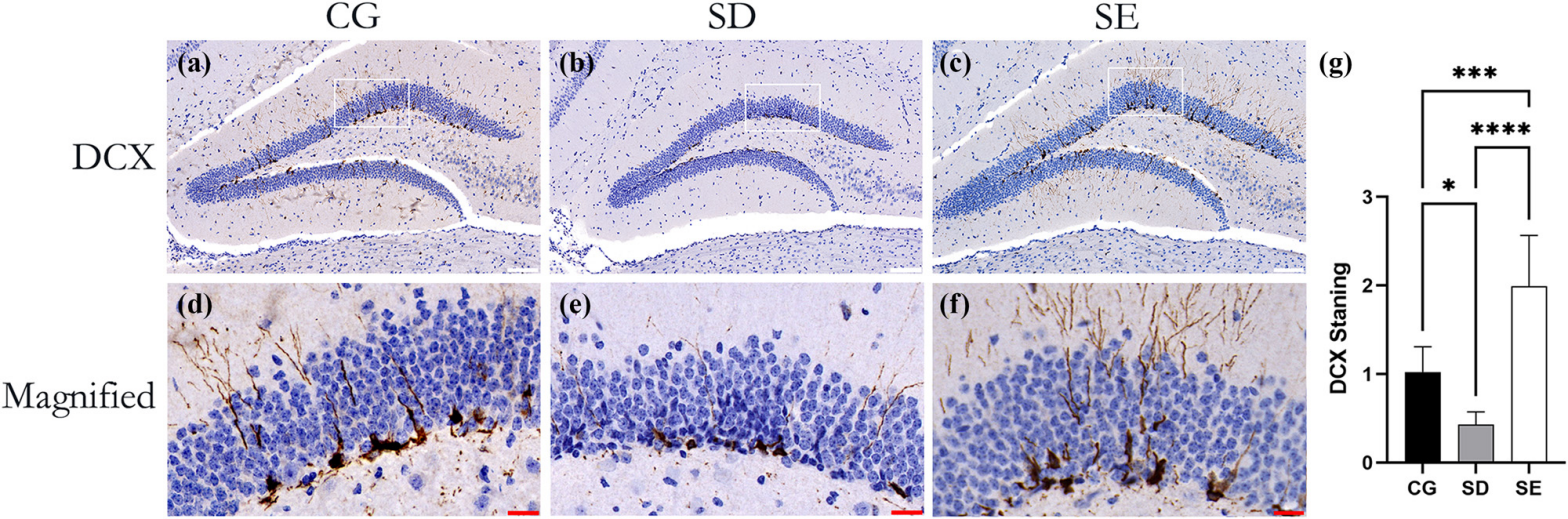
EEs reverse the damage of neurogenesis in DG under chronic sleep-deprived stimulation. DCX immunohistochemical staining showed that the expression in the SD group was decreased (*P* < 0.05) compared with the CG group. Compared with the SD group, the EE group’s expression significantly increased (*P* < 0.0001), even far more than that of the CG (*P* = 0.0001) (a–c, g). Under high magnification, it can be seen that DCX is mainly emitted from the junction of GCL and SGZ and extends to the GCL (d–f). Bars represent ± SEM; *n* = 8 mice per group; *P < 0.05; ***P < 0.001; ****P < 0.0001; CG = control group; SD = sleep deprivation; SE = sleep deprivation + enriched environment; DCX = double cortex element; GCL = granule cell layer; SGZ = subgranular zone; white scale bar = 100 μm, red scale bar = 20 μm.

There was no significant difference in DCX distribution, cell body outline, and synaptic morphology among the three groups. Compared with the CG, under long-term SD, neurogenesis was reduced by 57.9% ((*F* (2,21) = 34.64, *P* = 0.0136; Figure 3g). The EE reversed this process ((*F* (2,21) = 34.64, *P* < 0.0001; Figure 3g), and the expression of DCX exceeded even that of the CG by 48.7% ((*F* (2,15) = 34.64, *P* = 0.0001; Figure 3g).

### 3.2 EE reverses nerve cell loss from chronic SD

As shown earlier (Figure 3g), SD and environmental enrichment significantly affected neurogenesis in the DG region. However, since DCX is a transient marker of newborn neurons, the result of our observation may be the influence of SD and the environment at a specific moment [25]. To exclude this time factor, we used BrdU as a marker of neural precursor cells to examine the effect on the proliferation of neural stem cells in the DG area.

**Figure 4:**
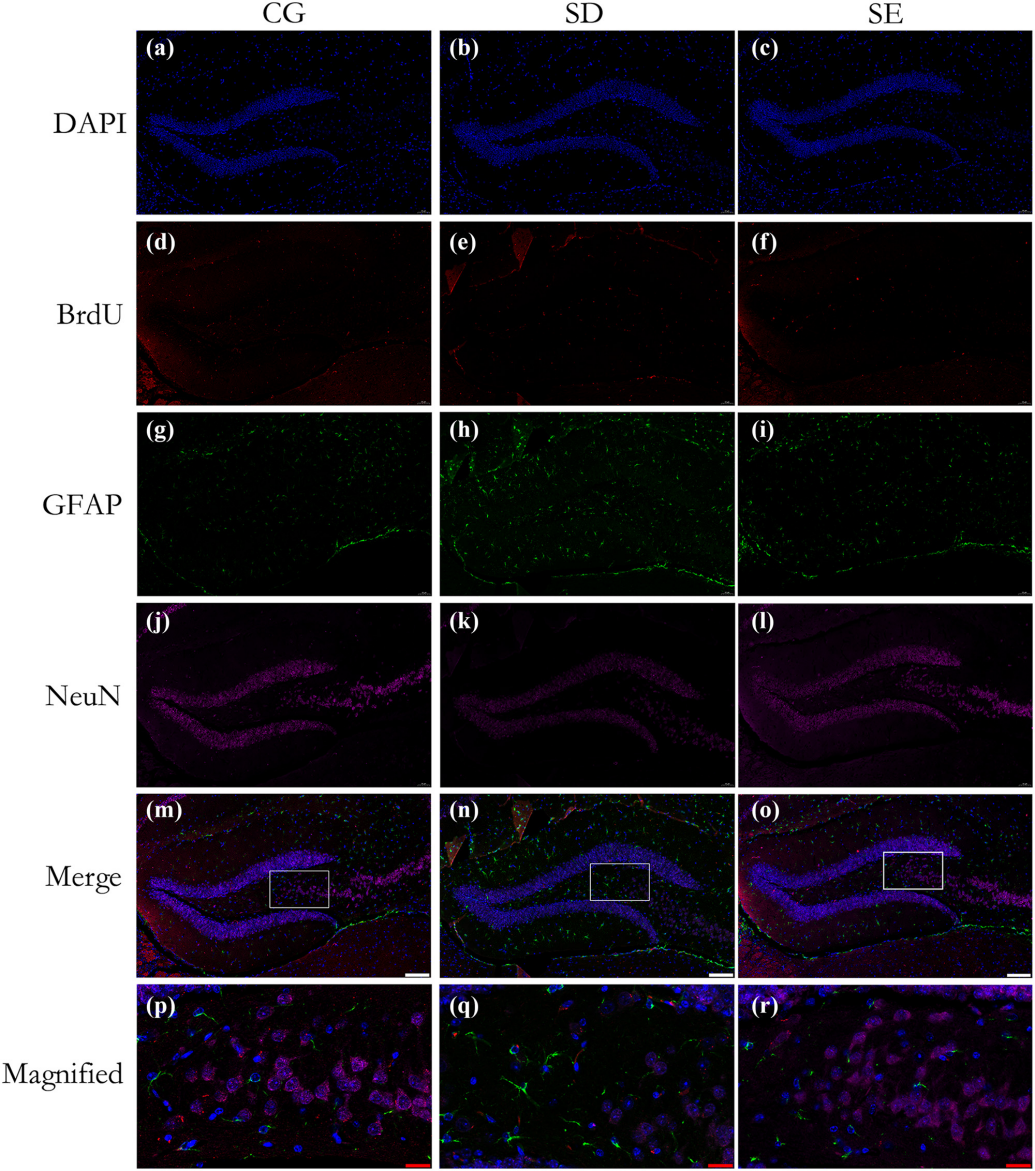

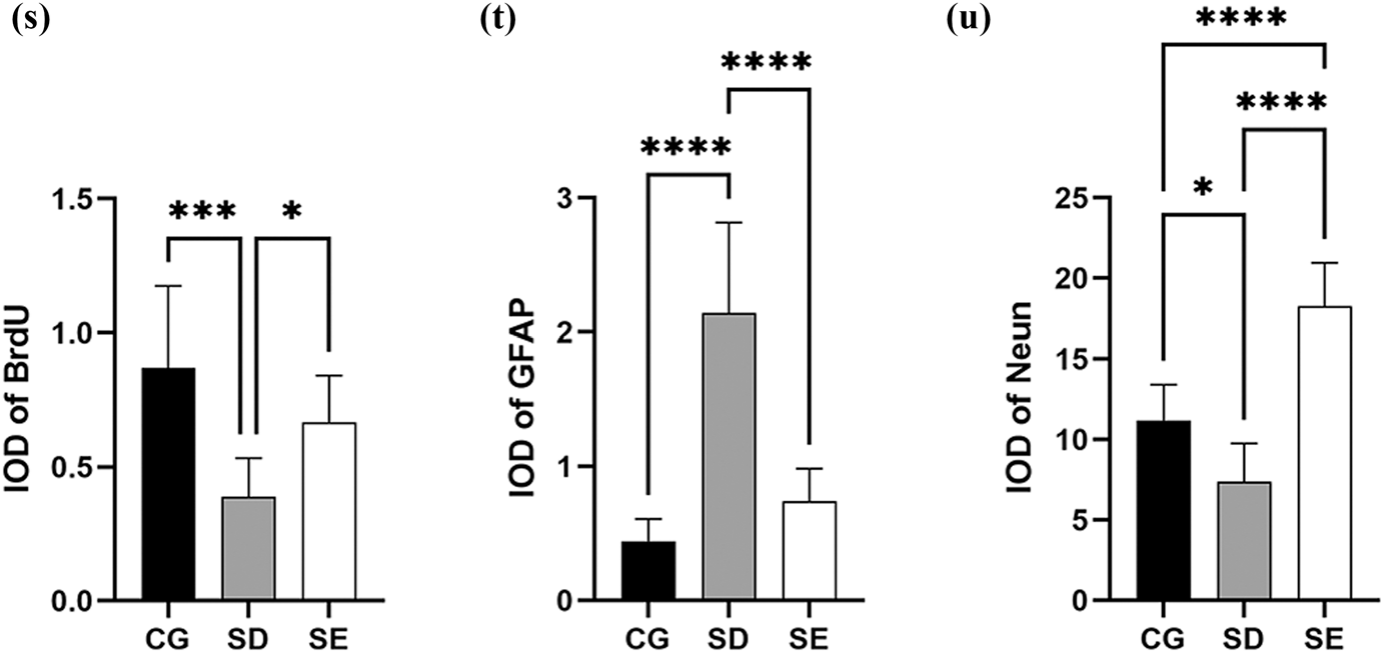
Enrichment stimulation reverses SD-induced decline in neuroplasticity in the DG. Triple immunofluorescence staining was used to label BrdU (neural progenitor cells) (red), GFAP (astrocyte cells) (green), NeuN (mature neurons) (pink), and DAPI (nuclei) (cyan). Compared with the CG, the expression level of BrdU in chronic SD mice was reduced by 55.3% (*P* < 0.001) (d, e, s). Compared with the SD group, the expression level of BrdU in the SE group increased by 41.8% (*P* < 0.05) and was still lower than that of the CG by 23.2% (*P* > 0.05) (e, f, s). Compared with the CG, the expression of NeuN in the DG region of the SD group decreased by 34.0% (*P* < 0.05) (j, k, u). Compared with the SD group, the SE group increased significantly and exceeded the CG by 38.8% (*P* < 0.0001) (j-l; u). Compared with the CG, the GFAP expression in the DG region of the SD group was significantly increased (79.3%; *P* < 0.0001) (g, h, t). The SE group decreased by 65.4% compared with the SD group (*P* < 0.0001) (h, i, t). There was no significant difference in GFAP expression between the CG and SE group (g, i, t). Bars represent ±SEM; *n* = 8 mice per group; *P < 0.05; ***P < 0.001; ****P < 0.0001; CG = control group; SD = sleep deprivation; SE = sleep deprivation + enriched environment; BrdU = 5-Bromo-2-deoxyuridine; GFAP = glial fibrillary acidic protein. White scale bar = 100 μm, red scale bar = 20 μm.

We stained sections of the DG marker region using triple immunofluorescence labeling. We compared the differences in the expression of neural precursor cells and their effects on differentiation into mature neurons and astrocytes. Compared with the CG, the expression level of BrdU in chronic SD mice was reduced by 55.3% ((*F* (2,21) = 9.716, *P* = 0.0007; Figure 4d, e, s). Compared with the SD group, the expression level of BrdU in the SE group increased by 41.8% ((*F* (2,21) = 9.716, *P* = 0.0471; Figure 4e, f, s) and was still lower than that of the CG by 23.2% ((*F* (2,21) = 9.716, *P* = 0.1813; Figure 4d and f, s). However, there was no statistical difference in the positive expression of BrdU in the DG region between the CG and SE group. Compared with the CG, the expression of NeuN in the DG region of the SD group decreased by 34.0% ((*F* (2,21) = 41.08, *P* = 0.0137; Figure 4j, k, u). Compared with the SD group, the SE group increased significantly by 59.6% ((*F* (2,21) = 41,08, *P* < 0.0001; Figure 4k, l, u) and exceeded the CG by 38.8% (*P* < 0.0001) (Figure 4j and l, u), which was a trend that was consistent with neurogenesis. These results show that under long-term environmental stimulation, the number of neurons in the DG area of the brain will increase significantly and even completely reverse the decline in neural repair ability caused by long-term SD.

### 3.3 EE can delay chronic SD-induced gliosis in the DG region of mice

Compared with the CG, the GFAP expression in the DG region of the SD group was significantly increased (79.3%, (*F* (2,21) = 36.66, *P* < 0.0001; Figure 4g, h, t). The SE group decreased by 65.4% compared with the SD group ((*F* (2,21) = 36.66, *P* < 0.0001; Figure 4h, i, t). Interestingly, there was no significant difference in GFAP expression between the CG and SE group ((*F* (2,21) = 36.66, *P* = 0.3563; Figure 4g and i, t). The result means that the SE group can delay the influence on the overexpression of GFAP caused by long-term SD to levels comparable to controls.

## 4 Discussion

### 4.1 EE reverses the impairment of neural stem cell proliferation and differentiation into mature neurons caused by long-term SD

Previous studies have shown that EE can improve cognitive function, anxiety, and depression behavior in sleep-deprived mice. The process relates to brain-derived neurotrophic factors, synaptic plasticity, and neurotransmitters [26–28]. However, the relationship between EE and cellular plasticity in chronic SD models remains unexplored. Given its role in neuronal differentiation and migration, DCX is currently recognized as a marker for newborn neurons [29]. In addition, we used triple immunofluorescence staining to test the proliferation and differentiation phenotypes of neural progenitor cells in the DG region of the hippocampus. BrdU replaces thymine for DNA synthesis during the S phase of cell division. Different from transient markers of neural progenitor cells such as Ki67, minichromosome maintenance protein 2, and phosphorylated histone 3, the BrdU represents the total number of neuronal cell proliferation during the injection period [25]. Since 80% of immature cells die within the first week after generation, BrdU represents the number of viable cells beyond this period but not neuronal cell proliferation [30]. In our experimental protocol, BrdU injection for five consecutive days can be more specific than the previous study to reflect the proliferation of neural progenitor cells during this period.

The isolated environment inhibits the brain’s proliferation of neural progenitor cells [21]. In our study, EE reversed the decrease in BrdU expression caused by chronic SD. Also, EE reversed neuron differentiation damage caused by chronic SD far beyond the CG. However, the amount of mature neurons is mainly affected by the proliferation and differentiation of neural progenitors and the survival of new neurons. We obtained a near-consistent trend between DCX and NeuN. It is related to the enhancement of the proliferation and differentiation ability of stem cells. It remains to be further studied if the survival rate of newborn neurons is the reason for our results.

Previous SD studies often selectively impair the REM period of sleep, which is a critical period for learning and memory formation [31]. Kreutzmann et al. believed that the cognitive impairment caused by REM loss was mainly related to intracellular cyclic adenosine monophosphate (cAMP)–protein kinase A (PKA) signaling and was often accompanied by a decrease in nerve cell proliferation and occurrence. The decline of neuronal plasticity is caused by cAMP–PKA signaling-mediated response element binding protein and glutamate receptor subunits phosphorylation pathway and activity damage [13]. In addition, the classic learning pathway of synaptic plasticity brain-derived neurotrophic factor (BDNF)/tyrosine receptor kinase (TrkB)/extracellular signal-regulated kinase signaling and laminin–PrPC interaction is also involved in neurogenesis [32,33]. In conclusion, many previous scholars believed that SD impairs the plasticity of hippocampal neurons, which is entirely consistent with our results.

Although there is a lack of research on EE and chronic SD, many researchers have reached relatively consistent conclusions regarding EE and cellular plasticity in transgenic AD mouse models. Herring et al. found that neural progenitor cells in the SGZ of β-amyloid precursor protein hemizygous transgenic (TgCRND8) mice in the enrichment were significantly increased to the level of wild-type mice in the standard environment [22]. Rodriguez et al. performed EE intervention on 3xTg (KM670/671NL) transgenic mice for 6 months. Compared with the running environment alone and the standard environment, the mice in the enrichment group had significantly increased neurogenesis in the hippocampal DG area [25]. In the same intervention, Levi and Michaelson compared the balance between neurogenesis and apoptosis in the hippocampus of human apoE3- and apoE4-transgenic mice and showed that the balance favored the latter [34]. This study showed that EE promoted the proliferation and differentiation of neural stem cells and accelerated the apoptosis of hippocampal neurons. Our study did not assess the apoptosis of hippocampal neurons. However, our findings on enhanced neuronal cell plasticity are consistent with previous studies. Future research must explore the molecular mechanism of EE promoting the plasticity of hippocampal neurons in mice with chronic SD.

### 4.2 EE can delay long-term SD-induced gliosis

Previous studies have shown that neurodegeneration is thought to be closely related to dementia in AD, including initial synaptic damage and subsequent neuronal loss, often accompanied by the proliferation of glial cells [35]. Neurogenesis in the hippocampal SGZ begins in neural progenitor cells. Most neural progenitor cells differentiate into dentate granule cells and eventually into mature neurons and then synaptically integrate into existing neural networks. The other small fraction differentiates into glial cells [36]. Glial cells can act as a physical barrier to the spread of tissue inflammation [37]. However, with the formation of a glial scar, excess gliosis impairs the ability of central nervous system nerve regeneration [38]. The latest research shows that astrocytes can promote myelin formation through neurotrophic factors such as BDNF and ciliary neurotrophic factor [39]. In addition, apolipoprotein E (APOE) is mainly expressed in nervous system astrocytes [40]. APOE, as a lipid transport carrier, can transport lipids to glial cells to promote myelin formation in neurogenesis. GFAP is an intermediate filament protein that constitutes the skeleton of cells and organelles and participates in the mechanosensing of the extracellular environment [41]. Overexpression of GFAP is often considered to be a marker of gliosis following long-term brain injury [42].

In our study, long-term SD caused a significant increase in the expression of GFAP, which is consistent with previous studies in AD transgenic mice. The conclusion suggests that the mechanism of long-term chronic SD causing AD may be related to the proliferation of glial cells. However, there is still some controversy about the relationship between EE and astrocytes. For example, in animal models of stroke and schizophrenia, EE increased the number of astrocytes in the hippocampus [43,44]. In AD mouse models, Beauquis et al. found that 3-month enrichment stimulation prevented astrocyte morphological changes [45]. This morphological change may be a compensatory mechanism by which astrocytes act on neurotrophic factors through TrkB T1 receptors after dendrite loss [46,47]. However, the living environment had no significant effect on the number of glial cell differentiation [22]. Our conclusion is similar to AD transgenic mice. EE does promote neurogenesis by reducing gliosis. In our experiment, the neurogenesis in the SE group was significantly increased, even far exceeding that in the CG. The expression of GFAP in the CG and SE group had no statistical difference, and the SE group was lower than the average value of the SD group. This trend, consistent with neurogenesis, also implied that the mechanism by which EE reverses neurogenesis might be related to glial cells. In conclusion, these studies all show that EE can promote nerve regeneration by inhibiting glial scar formation.

## 5 Conclusion

In conclusion, our study shows that chronic SD leads to a decrease in the plasticity of hippocampal cells, which is consistent with the conclusions of previous studies. Importantly, however, we are the first to apply a classic paradigm for studying brain plasticity, EE, to a model of chronic SD and evaluate the results. Our study broadens the application of EE, proves that EE can delay the decline of brain cell plasticity caused by chronic SD, and provides a new intervention for models of chronic SD.
